# Prevalence of hypertensive disorders of pregnancy in Ethiopia: a systemic review and meta-analysis

**DOI:** 10.1186/s12884-018-1667-7

**Published:** 2018-01-18

**Authors:** Abadi Kidanemariam Berhe, Getachew Mullu Kassa, Gedefaw Abeje Fekadu, Achenef Asmamaw Muche

**Affiliations:** 1 College of Medicine and Health science, Adigrat University, Tigray, Ethiopia; 2 College of Health Sciences, Debre Markos University, Debre Markos, Ethiopia; 3 School of Public Health, College of Medicine and Health Sciences, Bahir Dar University, P.O.Box 79, Bahir Dar, Ethiopia; 4 Department of Epidemiology and Biostatistics, Institute of public health, University of Gondar, Gondar, Ethiopia

**Keywords:** Prevalence, Hypertensive disorders of pregnancy, Preeclampsia, Eclampsia, Systemic review, Meta-analysis, Ethiopia

## Abstract

**Background:**

Although hypertensive disorders of pregnancy are the leading cause of poor perinatal outcomes in Ethiopia, there is no study that shows the national prevalence. Therefore, the aim of this study was to estimate the national pooled prevalence of hypertensive disorders of pregnancy from studies conducted in different parts of the country.

**Methods:**

Databases; MEDLINE, PubMed, HINARI, EMBASE, Google Scholar and African Journals Online were searched by using different search terms on HDP and Ethiopia. Joanna Briggs Institute Meta-Analysis of Statistics Assessment and Review Instrument was used for critical appraisal of studies. The analysis was done using STATA 14 software. The Cochran Q test and *I*^2^ test statistics were used to test heterogeneity of studies. Egger’s test was used to show the publication bias. The pooled prevalence of HDP and the odds ratio (OR) with 95% confidence interval was presented using forest plots.

**Result:**

Seventeen studies were included in this review, with a total of 258,602 pregnant women. The overall pooled prevalence of hypertensive disorders of pregnancy in Ethiopia was 6.07% (95% CI: 4.83%, 7.31%). The Subgroup analysis by region and year of study showed a higher prevalence of hypertensive disorders of pregnancy in Southern Nations, Nationalities, and Peoples’ Region, 10.13% (95% CI = (8.5, 12.43)), and reduction in the rate of HDP from 1990’s to 2010’s, 8.54% reducing to 5.71% respectively. The pooled prevalence of pregnancy-induced hypertension (PIH) and preeclampsia/eclampsia alone were 6.29 and 5.47 respectively. Pregnant women ≥ 35 years old are more likely to develop hypertensive disorders of pregnancy, OR = 1.64 (95% CI = (1.18, 2.28)). No statistically significant difference was observed between HDP and younger maternal age (less than 20 years old); OR = 2.92 (95% CI = (0.88, 9.70)). There was no association between hypertensive disorders of pregnancy and number of pregnancy, OR = 1.37 (95% CI = 0.78, 2.41)).

**Conclusions:**

The prevalence of hypertensive disorders of pregnancy is high in Ethiopia. The problem is more common among older pregnant women (> 35 years old). Government and other stakeholders should give due attention to an early screening of hypertension during pregnancy.

## Background

While motherhood is a positive and enjoyable experience, many women are experiencing suffering, illness, and death. Around 15% of pregnant women are expected to develop life-threatening complications during pregnancy, at delivery or post-partum. Hypertensive disorders of pregnancy(HDP) are significant contributors to these complications and sufferings [1–3].

A pregnant woman is considered hypertensive if her blood pressure is greater than or equal to 140/90 mmHg on two consecutive measurements [4]. Hypertensive disorders of pregnancy is a general term for increased blood pressure during pregnancy. It includes pregnancy-induced hypertension (PIH) (without proteinuria), preeclampsia (with proteinuria) and eclampsia (preeclampsia with convulsions), gestational hypertension and chronic hypertension [5].

Hypertensive disorders of pregnancy are public health problems globally. Global studies showed that preeclampsia and eclampsia were associated with higher rates of maternal mortality, prenatal mortality, and morbidity, preterm and small for gestational age deliveries. Women with HDP are five times more likely to have perinatal death compared with women who have no hypertensive disorders of pregnancy [6–9].

Pregnancy-induced hypertension complicates 10% of all pregnancies. Around 40,000 women, mostly from developing countries, die each year due to preeclampsia or eclampsia. Preeclampsia alone is estimated to account for about 40% to 60% of maternal deaths in developing countries [10–13]. A hospital-based study conducted in South Africa showed that HDP contributed for 20.7% of maternal deaths in the country [11].Hypertensive disorders of pregnancy accounts for 19% of maternal deaths in Ethiopia [14].

The Antenatal care (ANC) is one of the maternal care services in Ethiopia. Blood pressure measurement and urine tests for protein urea are among the components of routine ANC. According to the 2016 Ethiopian demographic and health survey (EDHS) report, 62% of pregnant women had at least one ANC visit. From this, 75% of pregnant women had their blood pressure measured and 66% had a urine test [11].

The prevalence of preeclampsia in developing countries ranges from 1.8% to 16.7% [10]. The incidence of HDP was estimated 9.8% in a study conducted in South Africa [11]. The prevalence of HDP in Ethiopia ranges from 1.2% to 18.25% according to various studies conducted [15–31].These studies are inconsistent and inconclusive to show the national magnitude. The country level estimate is essential to design evidence-based interventions. Therefore, this systematic review and meta-analysis was designed to estimate the national pooled prevalence of hypertensive disorders of pregnancy in Ethiopia and regions.

## Methods

### Study design and search strategy

A systemic review and meta-analysis was conducted from published and unpublished researches on the prevalence of hypertensive disorders of pregnancy in Ethiopia. The studies were retrieved through internet search from the databases of MEDLINE, PubMed, EMBASE, HINARI, Google Scholar and the African Journals Online (AJOL). The search was done using the following search terms; prevalence, hypertensive disorders of pregnancy, gestational hypertension, preeclampsia, eclampsia, Pregnancy-induced hypertension, and Ethiopia. The reference lists of already identified studies were screened to retrieve additional articles. All published articles up to16 December 2016 were included in this review. Unpublished studies were retrieved from Addis Ababa University electronic library and by requesting authors [32].

### Eligibility criteria

Studies were included in the review if the study;

1. design was cross-sectional, cohort, case-control or trial
2. was conducted on hypertensive disorders of pregnancy in Ethiopia
3. used the standard definition for classification of HDP (described in the outcome of interest)
4. was published in English

### Definition of outcomes of interest

- The primary outcome of this study is the prevalence of Hypertensive disorders of pregnancy. It is defined as a Systolic blood pressure (SBP)of 140 mmHg or more or diastolic blood pressure (DBP) of 90 mmHg or more on 2 or more consecutive occasions during pregnancy [33, 34].
- Eclampsia is defined as the occurrence of convulsions; DBP of 90 mmHg or higher after 20 weeks of pregnancy; proteinuria of 2+ or higher; and signs and symptoms of severe pre-eclampsia [19].
- Pre-eclampsia denotes for women who develop both hypertension and proteinuria in pregnancy [33].
- Gestational hypertension is defined as an elevation of DBP to 90 mmHg or more without proteinuria in a previously normotensive non-protein uric pregnant woman [33].
- Chronic hypertension in pregnancy is made on the finding of hypertension; at the first “booking visit” before the 20th week of pregnancy in the absence of trophoblastic disease or at any stage of pregnancy in women with proven chronic hypertension or which then persists more than 42 days after delivery [33].

The secondary outcome of interest of this review was association of HDP with maternal factors. These factors include; maternal age, which is categorized as young maternal age (less than 20 years), adult mothers (20–34 years old), and older mothers (aged ≥ 35 years) Gravidity in this review is categorized into; primigravida and multigravida. Primigravida is defined as women who are pregnant for the first time. Multigravida is women who are pregnant for two or more times.

### Data extraction

The data extraction was done using a tool developed by the 2014 Joanna Briggs Institute Reviewers’ Manual data extraction form [35]. The data extraction tool includes information on title, author, year of study, publication, study design, sample size, study participants, study area, response rate, sampling method, and definition used for HDP. Additionally, data collection tool for the number of HDP cases by maternal age (young and older mothers) and gravidity (primigravida and multigravida) was also included.

### Quality assessment and data collection

Joanna Briggs Institute Meta-Analysis of Statistics Assessment and Review Instrument (JBI-MAStARI) was used for critical appraisal of the studies. Retrieved published and unpublished studies were assessed for inclusion using their title and abstract. Then a full review of articles for quality assessment was done before selecting for final review. All authors independently assessed the articles for inclusion in the review. Any discrepancy which arose between the authors on the review process was solved through discussion.

### Publication bias and heterogeneity

The existence of heterogeneity was assessed using I^2^ and its corresponding P – value. A value of 25%, 50%, and 75% was used to declare the heterogeneity test as low, medium and high heterogeneity. For results with statistically significant heterogeneity, random effect model of analysis was used [36].Egger regression asymmetry test was used to assess the statistical significance of publication bias.

### Synthesis of result and statistical analysis

The data were entered using Microsoft Excel. The meta-analysis was conducted using Stata 14 software. Forest plots were used to present the combined estimate with the 95% confidence interval (CI). For studies which didn’t present standard error (SE), the formula; SE = √p x (1-p)/ n was used for calculation. The estimated pooled prevalence was computed with 95% CI. Subgroup analysis was done by region and type of HDP. Additionally, association of HDP with number of pregnancy and maternal age was conducted.

## Results

### Studies identified

**Fig. 1:**
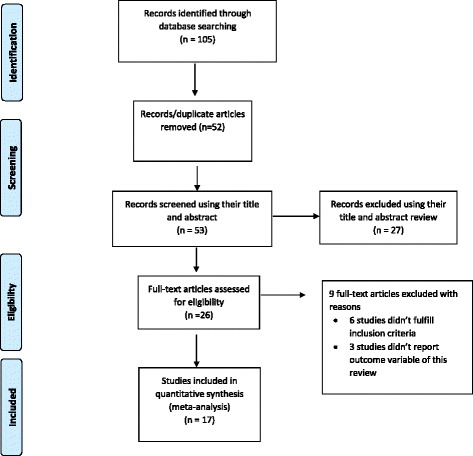
Flow diagram of the studies included in the Meta-analysis

A total of 104 articles were retrieved through electronic searching. In addition, one article was obtained by contacting authors through email, totally 105 articles were retrieved. Out of these, 52 duplicate records were removed from the review. Seventeen and ten articles were excluded by reviewing the title and abstract respectively. After a full review of articles, nine were excluded. Six studies didn’t fulfill the inclusion criteria and 3 articles fail to report the outcome variables. Finally, seventeen studies were included in this meta-analysis (Fig. 1).

### Description of studies

Thirteen of the included studies were Cross-sectional and four were case-control studies. Most of the regions in Ethiopia were represented in this review. Five studies were conducted in Addis Ababa city, five studies from Amhara region, two from Oromia region, three studies were conducted in SNNPR, one from Tigray and one was a nation based study. Studies were conducted from 1990 to 2016. The sample size of studies ranges from 291 a study conducted in Addis Ababa City(37)to a maximum of 174, 561, a nation based study [19]. Overall, this review included a total of 258,602 pregnant women in Ethiopia (Table 1).

**Table 1:** Summary of characteristics of studies included in the meta-analysis

S.No	Author/s	Studyyear	Study design	Sample size	SE	Study area	Prevalence of HDP (95% CI)
1.	Mekbeb and Ketsela	1991	Cross-sectional	6766	0.255	Yekatit 12 Hospital, Addis Ababa	5.14 [4.64, 5.64]
2.	Wolde et al.	2011	Cross-sectional	1863	0.646	Jimma University Specialized Hospital, Oromia Region	8.50 [7.23, 9.77]
3.	Terefe et al.	2015	Cross-sectional	8626	0.208	DebreBerhan Hospital, Amhara Region	3.90 [3.49, 4.31]
4.	Vata et al.	2015	Cross-sectional	7702	0.168	Dilla University Referral Hospital, SNNPR	2.23 [1.90, 2.56]
5.	Tessema et al.	2015	Cross-sectional	490	1.253	Dessie hospital, Amhara Region	8.40 [5.94, 10.86]
6.	Teklu and Gaym	2006	Cross-sectional	3424	0.382	TikurAnbessa Hospital, Addis Ababa	5.30 [4.55, 6.05]
7.	Hailu and Kebede	1991	Cross-sectional	576	1.374	Shiwa, Amhara Region	12.20 [9.51, 14.89]
8.	Wagnew et al.	2016	Cross-sectional	42,963	0.097	Government hospitals, Addis Ababa	4.20 [4.01, 4.39]
9.	Seyom et al.	2015	Cross-sectional	5415	0.208	Mettu Karl Hospital, Oromia Region	2.40 [1.99, 2.81]
10.	Selamawit D and Sisay T	2015	Cross-sectional	3488	0.438	Zewditu Memorial Hospital, Addis Ababa	7.20 [6.34, 8.06]
11.	Shambel W and Surender R	2016	Cross-sectional	320	1.58	Dessie referral Hospital, Amhara Region	8.80 [5.70, 11.90]
12.	Shegaze et al.	2016	Cross-sectional	422	1.88	Public Health Institutions in Arba Minch, SNNP Region	18.25[14.57, 21.93]
13.	Gaym et al.	2011	Cross-sectional	174,561	0.026	hospitals and health centers in Ethiopia	1.2[1.15,1.25]
14.	Teklit G et al.	2016	Case-control	291	–	ZewdituMemorial hospital and Gandhi Memorial hospital, Addis Ababa	–
15.	Yohannes T et al.	2016	Case-control	400	–	Tigray zonal Public Hospitals, Tigray Region	–
16.	Getinet A et al.	2016	Case-control	464	–	DerashieWoreda, SNNP Region	–
17.	Aklilu A et al.	2016	Case-control	831	–	BahrDar, Debre Markos, Gondar University hospitals, Amhara region	–

### Prevalence of HDP in Ethiopia

**Fig. 2:**
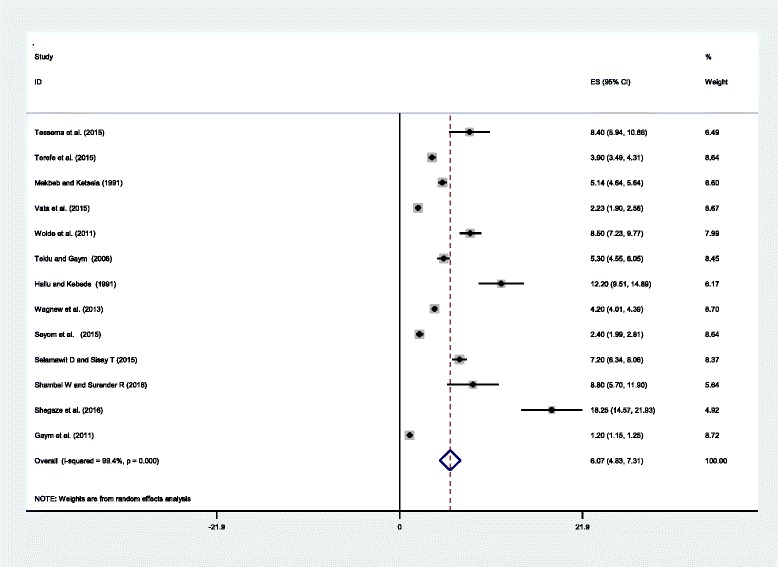
Forest plot displaying the pooled prevalence of Hypertensive disorders of pregnancy in Ethiopia

A wide-ranging prevalence of HDP was observed across the different studies included in this review. A prevalence of 1.2% in nation based study(19)to 18.25% in SNNPR were observed [25]. The *I*^*2*^test result showed high heterogeneity (I^2=^99.4%, *p*-value = < 0.001), which is indicative to use random effects model of analysis. So, using the random effect analysis, the overall pooled prevalence of hypertensive disorder of pregnancy in Ethiopia was 6.07% (95% CI: 4.83%, 7.31%) (Fig. 2).

### Subgroup analysis

Subgroup meta-analysis of the prevalence of HDP by region showed a higher pooled prevalence of HDP in SNNPR, 10.13% (95% CI = (8.5, 12.43)) and Amhara region, 8.21% (95% CI = (3.94, 12.48)). The lowest prevalence, 5.41% (95% CI = (4.30, 6.51)) was observed in Addis Ababa City, capital City of Ethiopia. The subgroup analysis by publication year showed a decrease in the prevalence of HDP from 1990’s to 2016in Ethiopia. Studies conducted during 1990 to 2000 showed a higher prevalence, 8.54% (95% CI = (1.63, 15.45) reducing to 5.71% (95% CI = (4.37, 7.06) during the year 2011 to 2016 (Table 2).

**Table 2:** Sub-group analysis of prevalence of HDP in Ethiopia by region and year of publication

Sub group	Number of studies included	Prevalence (95% CI)	Test of heterogeneity, I^2^	*p*-value
By region				
Addis Ababa City	4	5.41(4.30, 6.51)	94.8	< 0.001
Amhara region	4	8.21(3.94, 12.48)	94.6	< 0.001
Oromia region	2	5.42(3.7, 7.63)	98.8	< 0.001
SNNPR	2	10.13 (8.5, 12.43)	94.8	< 0.001
Nation based study	1	1.20(1.15, 1.25)	–	–
By publication year				
1990–2000	2	8.54(1.63, 15.45)	96.1	< 0.001
2001–2010	1	5.30(4.55, 6.05)	–	–
2011–2016	10	5.71(4.37, 7.06)	99.4	< 0.001

From the thirteen articles included for the prevalence study, seven assessed PIH while six studies showed the prevalence of HDP including chronic hypertension. The subgroup analysis by classification of HDP showed a prevalence of PIH, 6.29% (95% CI = (4.60,7.97) and HDP including chronic hypertension, 5.79%(95% CI = (4.07, 7.51). Further analysis of types of HDP also showed a pooled prevalence of pre-eclampsia/eclampsia of 5.47% (95% CI = (3.71, 7.22)(Table 3).

**Table 3:** Sub-group analysis of prevalence of HDP in Ethiopia by types of HDP

Sub group	Number of studies included	Prevalence (95% CI)	Test of heterogeneity, I^2^	*p*-value
By classification of HDP				
PIH	7	6.29 (4.60, 7.97)	99.5	< 0.001
Including Chronic HTN	6	5.79 (4.07, 7.51)	97.3	< 0.001
By type of HDP				
Pre-eclampsia and eclampsia	6	5.47 (3.71, 7.22)	99.6	< 0.001
Pre-eclampsia and gestational HTN	1	12.2 (9.51, 14.89)	–	–
All types of HDP	6	5.79 (4.07, 7.51)	97.3	< 0.001

### Association between maternal age and HDP

**Fig. 3:**
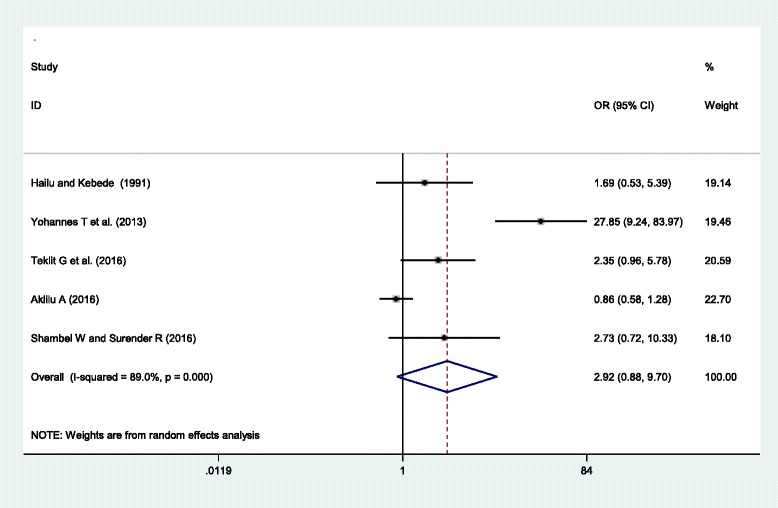
Forest plot displaying the association of young maternal age (less than 20 years old) and hypertensive disorders of pregnancy in Ethiopia

Five studies were included in this category of meta-analysis [20, 24, 37–39]. Only one of the included studies(38)showed a higher risk of HDP among young women (less than 20 years old) compared to the adult women (aged 20–34 years old). While four studies [20, 24, 37, 39] showed no difference in the rate of HDP between the two groups. The final pooled meta regression analysis showed no statistically significant difference in the rate of HDP between the young and adult women, OR = 2.92(95% CI = (0.88, 9.70)).High heterogeneity was observed in this category of meta-analysis, *I*^*2*^ = 89%, *P*-value = < 0.001. However, the test for publication bias using the Egger’s test showed non-statistical significant publication bias, *p*-value = 0.151 (Fig. 3).

**Fig. 4:**
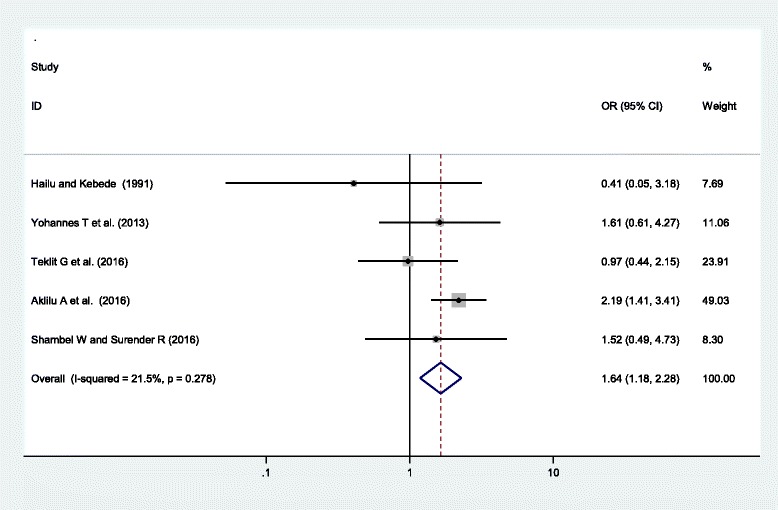
Forest plot displaying the association of older maternal age (≥ 35 years old) and hypertensive disorders of pregnancy in Ethiopia

This review also assessed the association of older maternal age (> = 35 years old) and adult women (aged 20–34 years old) with HDP. The pooled regression analysis result of five studies [20, 24, 37–39] showed a higher risk of HDP among older women compared to adult women, OR = 1.64 (95% CI = (1.18, 2.28)). The heterogeneity test of studies and test for publication bias using the Egger’s test showed non-significant heterogeneity (*I*^*2*^ = 21.5%, p-value = 0.278) and non-significant publication bias (p-value = 0.098) (Fig. 4).

### Gravidity and HDP

**Fig. 5:**
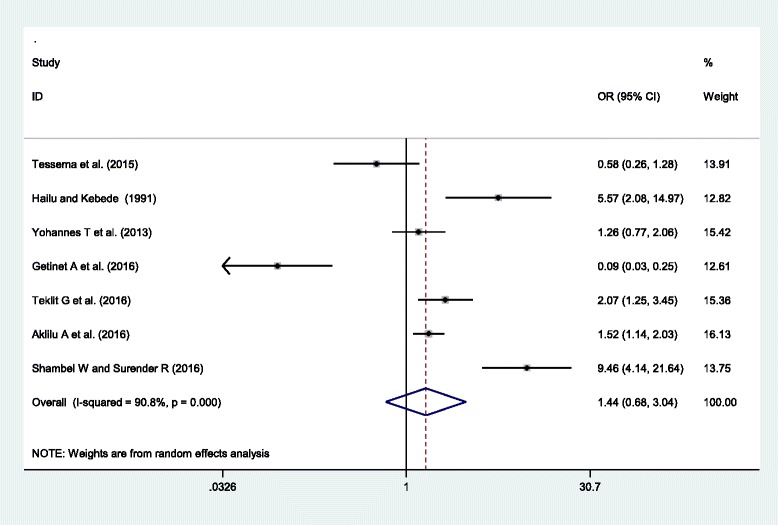
Forest plot displaying the association of primigravida and hypertensive disorders of pregnancy in Ethiopia

In this subcategory analysis, seven articles were included [20, 24, 28, 37–40]. Four of the included studies showed a statistically significant association of primigravida with HDP, higher risk of HDP among primigravida women than multigravida [20, 24, 37, 39]. While, one study [40] showed a lower risk of HDP among primigravida women and two studies [28, 38] showed non-significant difference between the two groups. The pooled meta-regression analysis showed that there is no statistically significant difference in the occurrence of hypertensive disorders of pregnancy in primigravidae and multigravida women, OR = 1.37 (95% CI = 0.78, 2.41)). The studies included in this analysis were heterogeneous as evidenced by *I*^*2*^ (*I*^*2*^ = 90.2%, *p*-value = < 0.001) and there is no publication bias as indicated by the Egger’s regression test (p-value> 0.883). Therefore, random effect model was used to estimate the effect of gravidity on hypertensive disorder of pregnancy (Fig. 5).

## Discussion

Hypertensive disorders of pregnancy are the commonest medical complication of pregnancy. The incidence varies in different populations. Generally, the problem is more common in the developing countries compared to developed countries. In Ethiopia, 19% of the maternal mortality is caused by HDP [14].

According to the results of this meta-analysis, the pooled prevalence of hypertension disorder of pregnancy in Ethiopia was estimated to be 6.25% (95% CI: 5.23%, 7.26%). Regional variation in HDP was observed, the highest prevalence of HDP (18.25%) was reported in a study done in Public Health Institutions in Arba Minch town, SNNPR [41].The lowest prevalence of HDP (1.2%) was observed in different hospitals and health centers from different regions of Ethiopia [15]. The variation in prevalence of HDP across region might be due to the difference in lifestyle like diet and physical activity and due to the difference in ANC service utilization across regions. Subgroup analysis findings showed that the prevalence of hypertensive disorders of pregnancy from 1990 up to 2000 and after 2011 was 8.54% and 5.71% respectively. This progress in the reduction of prevalence may be due to the improvement in health service coverage, expansion of health extension program and improvement in antenatal care coverage and services in the country.

The overall pooled prevalence of hypertensive disorders of pregnancy in this study is more or less similar to the large study conducted on HDP in China which was estimated 5.2% [42]. But, the finding is higher than the global prevalence [43, 44]. This difference might be due to socio-cultural, variability in maternal risk factor distribution, and the difference in antenatal care service accessibility. In addition, most of the studies included in this meta-analysis were conducted in hospitals and health centers which might increase the prevalence.

Young maternal age was not associated with HDP. Similar finding was also observed in a systematic review on pre-eclampsia. The study showed that young maternal age doesn’t affect the risk of developing pre-eclampsia [45]. But, other studies showed different findings in the occurrence of HDP among younger and older mothers. For example, studies conducted in Nigeria, Cameroon, and Brazil showed a higher risk of HDP among young women [46–48]. While, studies conducted in Ontario and South Glamorgan region of Wales showed a lower risk of HDP among young mothers than older women [49, 50]. The difference between this review and other studies could be attributed to the difference in sociodemographic characteristics and the difference in the age classification for the younger and adult/older maternal age group.

The finding of this meta-analysis also showed a significant association of hypertensive disorders of pregnancy with increasing age. Women aged more than 35 years old were 1.64 times more likely to develop HDP than women aged 20–34 during their pregnancy. The increasing risk of HDP in older mothers could be related to the abnormally high lipid profile, high-density lipid cholesterol, and higher risk of vascular damage in this age group compared to younger women [51, 52]. According to one of a study conducted in Addis Ababa, Ethiopia showed the most common Metabolic syndrome components among women using the ATP III criteria were High-Density Lipid concentration (23.2%) and abdominal obesity (19.6%) and it is higher among older women compared to young women [53]. A 4% rising risk in the rate of late pre-eclampsia and gestational hypertension for every year above the age of 32 years was also observed in a study conducted in United Kingdom [54].

A systematic review of controlled studies to assess the risk factors for pre-eclampsia at antenatal booking also showed that, women aged ≥40 were almost two times more likely to develop pre-eclampsia than younger women, irrespective of their previous history of pregnancy [45].Similar findings were also observed in studies conducted in United States, a review conducted by WHO, a study conducted in Asian population and in Latin America and Caribbean women [52, 55–58]. A survey on HDP in China also showed an increasing prevalence of HDP in pregnant women aged 40 years and older than pregnant women aged 25–29 years, 12.73% and 4.33% respectively [59].

Gravidity was not found associated with HDP in the current review. This finding is inconsistent with other studies [52, 59, 60]. The number of studies included in this meta-analysis is small. This may be the reason for the absence of an association between gravidity and HDP. The other reason may be the category of gravidity used in this study. Studies included in this review didn’t classify the gravidity into primigravida, multigravida, and grand multigravida. This classification may have masked the association. But, other studies showed that grand-multigravida is at increased risk of HDP [59].

A comprehensive search strategy was employed to retrieve both published and unpublished studies. PRISMA guideline was followed during the review process. This review assessed the association of maternal age with HDP. But, factors associated with HDP are not limited to maternal age and gravidity. So, further wide-scale studies and reviews are needed to assess the association of maternal socioeconomic and use of antenatal care with HDP.

## Conclusion

Even though there is a reduction in the rate of hypertensive disorders of pregnancy in the country, the rate of reduction is very low. HDP is still a common pregnancy-related disorder in Ethiopia. The problem is higher among older women aged ≥ 35 years old compared adult women aged 20 to 34 years old. While young maternal age showed no association with the occurrence of HDP. Gravidity was also not associated with HDP.

Therefore, early detection and treatment for HDP are needed for pregnant mothers aged ≥ 35 years old. Further study to assess the association between gravidity and HDP in Ethiopia are also needed. Government and other stakeholders should give due attention to early screening and treatment of HDP. Community based approaches to diagnose and treat the problem is recommended. Further nation based studies and reviews are needed to show the association of HDP with other socio-demographic, maternal and health service related factors.

## Abbreviations

ANC: Antenatal care
ATP: Adenosine Triphosphate
CI: Confidence Interval
EDHS: Ethiopian Demographic and Health Survey
HDP: Hypertensive Disorders of Pregnancy
JBI-MAStARI: Joanna Briggs Institute Meta-Analysis of Statistics Assessment and Review Instrument
OR: Odds ratio
PIH: Pregnancy induced hypertension
SE: Standard Error
SNNPR: Southern Nations, Nationalities, and Peoples’ Region of Ethiopia
WHO: World Health Organization
